# Supplementary material for: Causal Relationship between Aging and Anorexia Nervosa: A White-Matter-Microstructure-Mediated Mendelian Randomization Analysis
Source: Biomedicines. 2024 Aug 16;12(8):1874. doi: 10.3390/biomedicines12081874 (PMC11351342; doi:10.3390/biomedicines12081874)
Supplement: Supplementary file 1 [file biomedicines-12-01874-s001.zip › Supplementary Figure/Figure S4/Singe SNP plot of MR analysis for TL to ubm-b-1993.pdf]

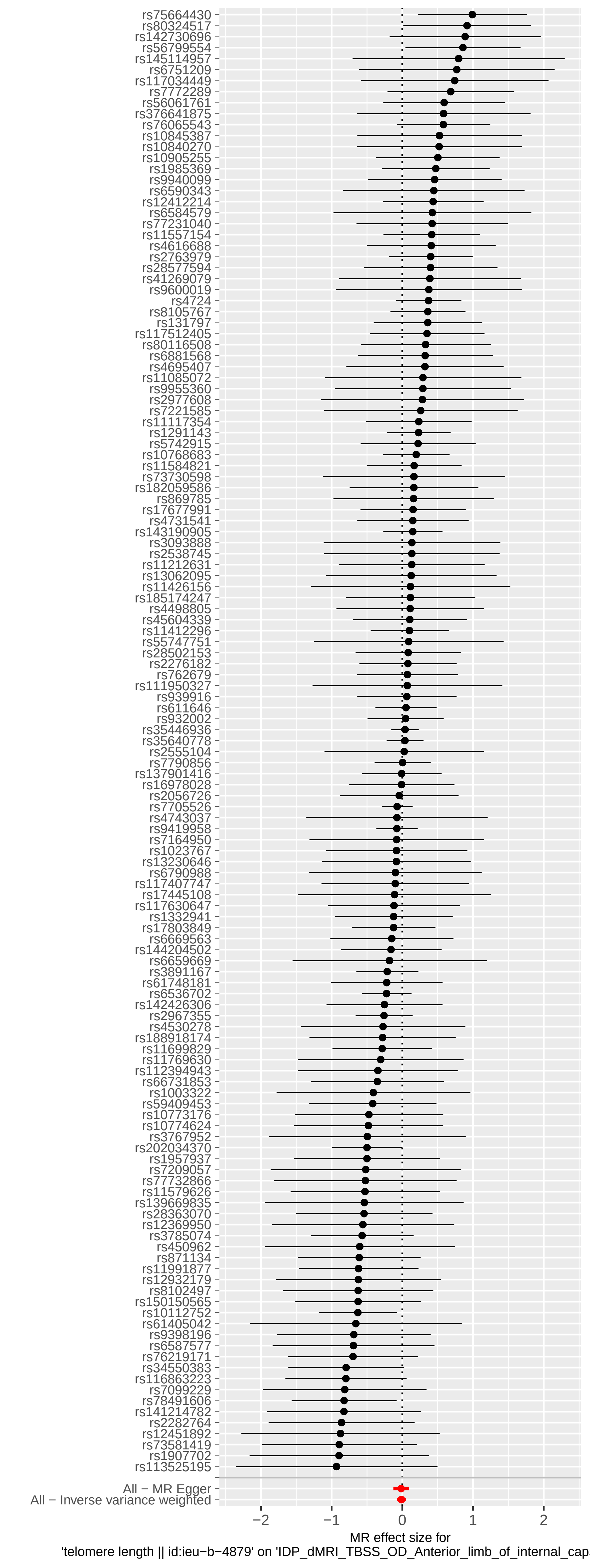
rs75664430
rs80324517
rs142730696
rs56799554
rs145114957
rs6751209
rs117034449
rs7772289
rs56061761
rs376641875
rs76065543
rs10845387
rs10840270
rs10905255
rs1985369
rs9940099
rs6590343
rs12412214
rs6584579
rs77231040
rs11557154
rs4616688
rs2763979
rs28577594
rs41269079
rs9600019
rs4724
rs8105767
rs131797
rs117512405
rs80116508
rs6881568
rs4695407
rs11085072
rs9955360
rs2977608
rs7221585
rs11117354
rs1291143
rs5742915
rs10768683
rs11584821
rs73730598
rs182059586
rs869785
rs17677991
rs4731541
rs143190905
rs3093888
rs2538745
rs11212631
rs13062095
rs11426156
rs185174247
rs4498805
rs45604339
rs11412296
rs55747751
rs28502153
rs2276182
rs762679
rs111950327
rs939916
rs611646
rs932002
rs35446936
rs35640778
rs2555104
rs7790856
rs137901416
rs16978028
rs2056726
rs7705526
rs4743037
rs9419958
rs7164950
rs1023767
rs13230646
rs6790988
rs117407747
rs17445108
rs117630647
rs1332941
rs17803849
rs6669563
rs144204502
rs6659669
rs3891167
rs61748181
rs6536702
rs142426306
rs2967355
rs4530278
rs188918174
rs11699829
rs11769630
rs112394943
rs66731853
rs1003322
rs59409453
rs10773176
rs10774624
rs3767952
rs202034370
rs1957937
rs7209057
rs77732866
rs11579626
rs139669835
rs28363070
rs12369950
rs3785074
rs450962
rs871134
rs11991877
rs12932179
rs8102497
rs150150565
rs10112752
rs61405042
rs9398196
rs6587577
rs76219171
rs34550383
rs116863223
rs7099229
rs78491606
rs141214782
rs2282764
rs12451892
rs73581419
rs1907702
rs113525195
All − MR Egger
All − Inverse variance weighted
−2
−1
0
1
2
MR effect size for
'telomere length || id:ieu−b−4879' on 'IDP_dMRI_TBSS_OD_Anterior_limb_of_internal_caps
